# Oxysterols and Their Cellular Effectors

**DOI:** 10.3390/biom2010076

**Published:** 2012-02-15

**Authors:** Vesa M. Olkkonen, Olivier Béaslas, Eija Nissilä

**Affiliations:** 1 Minerva Foundation Institute for Medical Research, Biomedicum 2U, Tukholmankatu 8, FI-00290 Helsinki, Finland; Email: olivier.beaslas@helsinki.fi (O.B.); eija.nissila@helsinki.fi (E.N.); 2 Institute of Biomedicine, Anatomy, Biomedicum 1, Haartmaninkatu 8, FI-00014 University of Helsinki, Finland

**Keywords:** cell signaling, EBI2, Insig, lipid metabolism, LXR, OSBP, oxysterol, oxysterol-binding protein, oxysterol receptor, ROR

## Abstract

Oxysterols are oxidized 27-carbon cholesterol derivatives or by-products of cholesterol biosynthesis, with a spectrum of biologic activities. Several oxysterols have cytotoxic and pro-apoptotic activities, the ability to interfere with the lateral domain organization, and packing of membrane lipids. These properties may account for their suggested roles in the pathology of diseases such as atherosclerosis, age-onset macular degeneration and Alzheimer’s disease. Oxysterols also have the capacity to induce inflammatory responses and play roles in cell differentiation processes. The functions of oxysterols as intermediates in the synthesis of bile acids and steroid hormones, and as readily transportable forms of sterol, are well established. Furthermore, their actions as endogenous regulators of gene expression in lipid metabolism via liver X receptors and the Insig (insulin-induced gene) proteins have been investigated in detail. The cytoplasmic oxysterol-binding protein (OSBP) homologues form a group of oxysterol/cholesterol sensors that has recently attracted a lot of attention. However, their mode of action is, as yet, poorly understood. Retinoic acid receptor-related orphan receptors (ROR) α and γ, and Epstein-Barr virus induced gene 2 (EBI2) have been identified as novel oxysterol receptors, revealing new physiologic oxysterol effector mechanisms in development, metabolism, and immunity, and evoking enhanced interest in these compounds in the field of biomedicine.

## 1. Introduction

**Figure 1:**
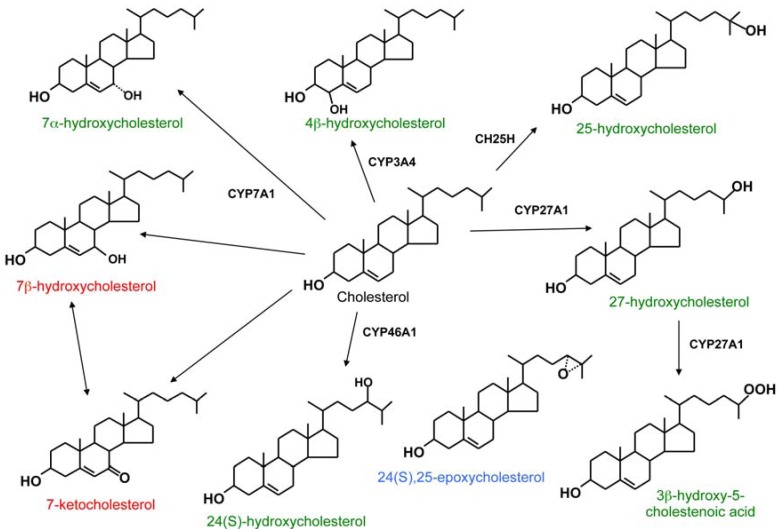
Structure and origin of selected common oxysterols. Most of the oxysterol species displayed are generated by enzymes that belong to the cytochrome P450 family (CYP). CH25H, cholesterol 25-hydroxylase, is a di-iron enzyme. The enzymatically derived species are indicated with green, products of cholesterol autoxidation with red, and a species derived from a shunt of the cholesterol biosynthetic process with blue print (Modified from [2] with kind permission from Springer Science+Business Media B.V.).

Oxysterols are 27-carbon oxidized derivatives of cholesterol or by-products of the cholesterol biosynthetic process with multiple biological activities. Several major oxysterols arise as intermediates in the pathways converting cholesterol to bile acids or steroid hormones, and their roles as readily transportable forms of sterol are well established [1,2,3]. The common oxygen-containing modifications of cholesterol in oxysterols are hydroxyl, keto, hydroperoxy, epoxy, and carboxyl moieties. In general, oxysterols have a drastically shorter biologic half-life than cholesterol—they can therefore be considered a way to route the cholesterol molecule for catabolism. The physiologically most important oxysterols are generated in cells by mitochondrial or endoplasmic reticulum cholesterol hydroxylases belonging to the cytochrome P450 family [4,5]. Of these species, the most abundant in human serum are 27-, 24(S)-, 7α-, and 4β-hydroxycholesterol (OHC). Of these, 24(S)-OHC originates from neurons in the central nervous system, the sterol homeostasis of which depends on the synthesis of this oxysterol catalyzed by the cholesterol hydroxylase CYP46A1 [6], 7α- and 27-OHC are synthesized by the liver by CYP7A1 and CYP27A1 as the first intermediates of classic and acidic bile acid synthetic pathways, respectively [7]; CYP27A1 however is functional also in non-hepatic cells [8]. 4β-OHC is generated by the hepatic drug metabolizing enzyme CYP3A4, which is markedly induced by certain anti-epileptic pharmaceuticals [9]. Oxysterols also arise *in vivo* or during food processing through non-enzymatic, free radical, lipid peroxide, or divalent cation-induced oxidative processes, often termed cholesterol autoxidation [10]. The most abundant oxysterols generated through autoxidation are modified at the 7-position of the cholesterol B-ring. These include 7-ketocholesterol (7-KC) and 7β-OHC with prominent cytotoxic and pro-apoptotic properties [11]. Structures of the most abundant oxysterols and the routes of their synthesis are depicted in Figure 1 [2], and the nomenclature of these oxysterols is specified in Table 1.

**Table 1:** Nomenclature of the oxysterols [3] discussed in this review.

Abbreviation	Common name	IUPAC name
β-EPOX	5β,6β-epoxycholesterol	Cholestan-5β,6β-epoxy-3β-ol
α-TRIOL		Cholestan-3β,5α,6β-triol
4β-OHC	4β-hydroxycholesterol	Cholest-5-en-3β,4β-diol
7α-OHC	7α-hydroxycholesterol	Cholest-5-en-3β,7α-diol
7β-OHC	7β-hydroxycholesterol	Cholest-5-en-3β,7β-diol
7-KC	7-ketocholesterol	Cholest-5-en-3β-ol-7-one
25-OHC	25-hydroxycholesterol	Cholest-5-en-3β,25-diol
27-OHC	27-hydroxycholesterol	(25*R*)-cholest-5-en-3β,26-diol
22(R)-OHC	22(R)-hydroxycholesterol	(22*R*)-cholest-5-en-3β,22-diol
20(S)-OHC	20(S)-hydroxycholesterol	(20*S*)-cholest-5-en-3β,20-diol
24(S)-OHC	24(S)-hydroxycholesterol	(24*S*)-cholest-5-en-3β,24-diol
24(S),25-EPOX	24(S),25-epoxycholesterol	(24*S*,25)-epoxycholest-5-en-3β-ol
7α,25-OHC	7α,25-hydroxycholesterol	Cholest-5-en-3β,7α,25-triol

Oxysterols are present in mammalian tissues at very low concentrations, as mixtures accompanied by a high excess of cholesterol. However, they are found enriched in pathologic structures such as macrophage foam cells, atherosclerotic lesions, cataracts, and gall stones. The cytotoxic and pro-apoptotic actions of oxysterols are suggested by play a role in the disease processes involved [12,13,14,15]. Moreover, oxysterols have been implicated in the pathology of degenerative diseases such age-onset macular degeneration and Alzheimer’s disease [16,17]. The above findings, as well as the potent regulatory activity that several oxysterols have on cellular cholesterol homeostatic machineries (reviewed by Gill *et al.* [18]), have prompted intensive research of oxysterols in the context of lipid homeostasis and atherosclerosis. However, novel physiologic activities of these compounds have emerged. Oxysterols are believed to act as endogenous regulators of gene expression in lipid metabolism and as signaling molecules with key roles in developmental, differentiation, and inflammation processes. Recently, novel oxysterol receptors responsible for these activities have been identified, opening new perspectives to oxysterol function and evoking novel interest in these compounds in the field of biomedical research. The present review focuses on the newly identified receptors and cellular effector pathways of oxysterols.

## 2. Oxysterols in Transcriptional Control of Lipid Metabolism

### 2.1. Oxysterols Act as Ligands of Liver X Receptors

The pioneering work by D. Mangelsdorf’s group revealed that the orphan nuclear receptors liver X receptor (LXR) α (NR1H3) and β (NR1H2) are activated by oxysterol ligands [19]. The physiologically most important endogenous LXR ligands are most likely 24(S),25-EPOX, 24(S)-OHC, 22(R)-OHC, 20(S)-OHC, and 27-OHC [19,20,21]. LXRα is expressed at highest amounts in liver, but also in adipose tissue, intestine, kidney, and macrophages, while LXRβ is ubiquitously expressed. The LXRs form heterodimers with retinoid X receptor (RXR) and play central roles in sterol absorption in the intestine, the reverse cholesterol transport process, bile acid synthesis, biliary neutral sterol secretion, hepatic lipogenesis, and synthesis of nascent high-density lipoproteins [22]—the LXRs have also been established as suppressors of inflammatory gene expression in macrophage [23,24], and recent studies have established an increasing number of LXR functions in immune regulation (see *3.4*.). Studies employing atherosclerosis-prone mouse models have revealed anti-atherogenic function of the LXRs [25,26]. However, LXR stimulation with synthetic agonists such as T0901317 also leads, via a lipogenic activity, to hepatic steatosis and increased serum triglyceride levels, which represents an atherosclerosis risk factor [27]. This caveat can be avoided via the use of intestine-specific LXR activation leading to reduced cholesterol absorption, as demonstrated by genetic and pharmacologic approaches [28,29]. While there is some controversy as to whether oxysterols truly act as endogenous ligands of the LXR [1]: The work by Chen *et al.* [30] provided evidence that this indeed is the case: Overexpression of an oxysterol catabolic enzyme, cholesterol sulfotransferase, was shown to inactivate LXR signaling in several cultured mammalian cell lines but did not alter receptor response to the nonsterol LXR agonist T0901317. Moreover, triple-knockout mice deficient in the biosynthesis of three oxysterol ligands of LXRs, 24(S), 25-, and 27-OHC, responded to dietary T0901317 by inducing LXR target genes but showed impaired responses to dietary cholesterol, supporting the view that conversion of cholesterol to oxysterols is important for LXR activation *in vivo*. On the other hand, the recent report by Shafaati *et al.* [31] showed that overexpression of CYP46A1 in transgenic mice under the β-actin promoter, resulting in significantly elevated 24(S)-OHC levels, failed to induce LXR target genes in either brain or liver. This data argues against a role of 24(S)-OHC, which is *in vitro* a potent LXR activator, as an important endogenous LXR agonist.

### 2.2. Oxysterols Regulate SREBP Maturation

The cellular machinery for cholesterol biosynthesis and uptake, as well as for fatty acid biosynthesis, is controlled by transcription factors designated sterol regulatory element binding proteins (SREBPs) [32,33]. The intracellular localization and proteolytic maturation of SREBPs are regulated by a cholesterol-sensor protein called the SREBP cleavage activating protein (SCAP). The SREBPs are synthesized as precursors anchored to ER membranes and form complexes with SCAP. Under low-cholesterol conditions, SREBP-SCAP complexes are transported by a coat protein complex II (COPII)-dependent mechanism to the Golgi apparatus, where SREBPs are proteolytically processed to release a basic helix-loop-helix leucine zipper transcription factor, which enters the nucleus and binds to sterol regulatory elements (SRE) in the promoters of target genes. When sterol builds up in cells, SCAP senses cholesterol in the ER membranes and interacts with Insig (Insulin-induced gene) proteins, and as a result the SREBP-SCAP complex is retained in the ER. The transport of SREBP to the Golgi complex is sensitive to both cholesterol and several oxysterols, of which 25-OHC is most commonly used in experimental set-ups. While SCAP senses ER cholesterol, it was not found to bind 25-OHC [34,35], raising the question of how the inhibitory effect of oxysterols could be mediated. Intriguingly, the Insig proteins were found to directly bind 25-OHC and to mediate the regulatory effect of this oxysterol on SREBP processing. Binding of the Insig-25-OHC complex to SCAP elicits a conformational change similar to that induced by cholesterol binding to SCAP. This precludes the interaction of SCAP with COPII proteins and thereby the transport and proteolytic activation of SREBP [36,37]. Interestingly, Lange *et al.* [38] found that the mitochondrial cholesterol 27-hydroxylase (CYP27A1) is required for the rapid inactivation of 3-hydroxy-3-methylglutaryl coenzyme A reductase (HMGCoAR) in response to high levels of cholesterol. This is most likely due to 27-OHC interaction with Insig, resulting in the ubiquitination and degradation of HMGCoAR (see [33]). Vertebrates thus have two sterol sensors that control SREBP activity, enabling cells to down-regulate their sterol biosynthesis upon the build-up of either cholesterol or its oxidized derivatives. 

24(S),25-epoxycholesterol [24(S),25-EPOX] is generated as a side product of the cholesterol biosynthetic process by the same enzymes that catalyze the synthesis of cholesterol [39]. 24(S),25-EPOX is a potent feedback regulator of cholesterol biosynthesis, suppressing SREBP-2 processing [40] and the cellular HMGCoAR activity [41]. It is also a highly potent oxysterol activator of the LXRs [20,30,42]. The cellular level of 24(S),25-EPOX can be manipulated either by using inhibitors of the cholesterol biosynthetic pathway enzyme 2,3-oxidosqualene cyclase (OSC), resulting in elevation of cellular 24(S),25-EPOX levels, or by overexpressing this enzyme to reduce the cellular content of the oxysterol. Using these tools the groups of A. Brown and M. Huff [43,44,45,46,47] have significantly added to our understanding of the physiologic role of 24(S),25-EPOX. Beyea *et al.* [43] demonstrated that partial inhibition of OSC in THP-1 macrophages reduced cholesterol synthesis and increased the expression of several LXR target genes, *ABCA1*, *ABCG1*, and *APOE*. Importantly, OSC inhibition did not stimulate lipoprotein lipase (*LPL*) or fatty acid synthase (*FAS*), and the observed induction of the lipogenic transcription factor *SREBP-1c* was counteracted by a block in its conversion to the active nuclear form, supporting the notion that OSC inhibition might have therapeutic potential [44]. However, this idea is undermined by the finding that 24(S),25-EPOX strongly inhibits cholesterol efflux from macrophage foam cells, possibly due to inhibition of cholesterol ester hydrolase function [48]. The findings of Wong *et al.* [45,46] suggest that synthesis of 24(S),25-EPOX parallels that of cholesterol and fine-tunes the acute control of cellular cholesterol homeostasis, thus protecting cells against accumulation of newly synthesized cholesterol. 

Interestingly, 24(S),25-EPOX is also suggested to have a specific role in CNS sterol homeostasis. Wong *et al.* [47] demonstrated that this oxysterol is produced by astrocytes and is taken up by neurons in which it impacts gene expression. The authors suggested that 24(S),25-EPOX may act as a signal from astrocytes that reduces the energetically costly cholesterol biosynthesis by neurons, enabling the neurons to direct resources to other processes essential for neurotransmission.

### 2.3. Oxysterols Modulate the Activity of RORα and γ

The retinoic acid receptor-related orphan receptors (RORs) are nuclear receptors that, as one of their physiologic functions, have been implicated in the transcriptional control of lipid metabolism. RORα (NR1F1) plays an essential role in development of the cerebellum and in regulation of the circadian rhythm [49], while RORγ (NR1F3) is best known for its role in regulation of T cell development [50]. Both RORα and RORγ are expressed in the liver and play roles in the regulation of glucose and lipid metabolism [51]. The group of T. Burris discovered in 2010 that the 7-substituted oxysterols bound to the ligand binding domains (LBDs) of RORα and RORγ with high affinity, altered the LBD conformation and reduced coactivator binding resulting in suppression of the constitutive transcriptional activity of these two receptors [52]. They recently reported that the two RORs also bind 24(S)-OHC (cerebrosterol), a brain-derived oxysterol, with high affinity, and that 24(S)-OHC functions as a RORα/γ inverse agonist suppressing the constitutive transcriptional activity of these receptors in cotransfection assays [53]. The authors also noted that 24(S),25-EPOX selectively suppressed the activity of RORγ. Interestingly, Wada *et al.* [54] found that RORα up-regulates expression of CYP7B1, which encodes oxysterol 7α-hydroxylase, an enzyme with a crucial role in bile acid synthesis and cholesterol metabolism. The authors also discovered that LXR target genes are induced in RORα-knock out mice and *vice versa*, suggesting a mutually suppressive function of these nuclear receptors. These findings indicate that RORα and RORγ serve as novel sensors for oxysterols and display an overlapping ligand preference and functional cross-talk with the LXR, suggesting the presence of an intriguingly complex sterol-controlled network of gene regulatory actions related with metabolic disease and atherosclerosis.

## 3. Oxysterols in Signaling and Development

### 3.1. Modulation of Hedgehog Signaling by Oxysterols

The Hedgehog (Hh) signaling pathway plays a central role in the patterning of metazoan embryos, in post-embryonic development, as well as in the homeostasis of adult tissues and stem cell physiology [55,56]. In 2006, the first evidence suggesting that cholesterol or certain oxysterols are required for Sonic hedgehog pathway signal transduction and proliferation of medulloblastoma cells was published by Corcoran and Scott [57]. Dwyer *et al.* [58] soon demonstrated that the oxysterols 20(S)- and 22(S)-OHC exert osteoinductive effects through activation of the Hh signaling pathway. Consistently, Kim *et al.* [59] provided evidence that the inhibition of bone marrow stromal cell differentiation into adipocytes by 20(S)-OHC occurs through a Hh-dependent mechanism, and later similar results have been reported with structural analogues of 20(S)-OHC [60]. In addition to activating the Hh signaling pathway, oxysterol-induced osteogenic differentiation was reported to be mediated through a Wnt signaling-related mechanism [61]. Furthermore, 20(S)-OHC was shown to activate Notch target genes, but apparently via a non-canonical mechanism, which may involve the LXR [62]. These findings have established a novel role of oxysterols as regulators of mammalian development. The recent report by Nachtergaele *et al.* [63] addresses the so far unidentified molecular mechanisms mediating the oxysterol impact on Hedgehog signaling: They found that the most potent oxysterol, 20(S)-OHC, acts as an allosteric activator of the trans-membrane protein Smoothened, which mediates the signal upon induction by Hedgehog ligands.

### 3.2. 27-Hydroxycholesterol Modulates Estrogen Receptor Function

Estrogen receptors are nuclear receptors that, in addition to central functions in reproductive biology, mediate estrogen regulation of a number of other physiologic processes [64]. The cardiovascular protection observed in pre-menopausal females has been largely attributed to beneficial effects of estrogen on endothelial function and the lipid profile [65,66]. Umetani *et al.* [67] discovered that 27-OHC antagonizes the estrogen-dependent production of NO by vascular cells, resulting in reduced vasorelaxation of aorta, a potentially deleterious effect. Moreover, increasing 27-OHC levels repressed carotid artery re-endothelialization. Also cell type-specific pro-estrogenic actions of 27-OHC were reported [67,68], indicating that this oxysterol acts as an endogenous selective estrogen receptor modulator (SERM). Through its actions on both estrogen receptors and liver X receptors, 27-OHC decreases osteoblast differentiation and enhances osteoclastogenesis, resulting in increased bone resorbtion in mice [69,70]. Furthermore, the action of 27-OHC on estrogen receptors has been putatively linked with breast cancer: A decrease in the expression of E-cadherin and β-catenin, paralleling the loss of adherens junction complex, was observed in MCF7 breast cancer cells exposed 27-OHC, indicating an epithelial-mesenchymal transition characteristic of tumor development [71]. The findings suggest that 27-OHC may counteract the estrogen protection from vascular disease, impair the bone homeostasis, and possibly modify oncogenesis in estrogen receptor-dependent cancers, thus revealing novel, potentially harmful oxysterol functions. 

## 4. Oxysterol-Induced Cell Death

Oxysterols are incorporated into biological membranes, and different oxysterols have distinct impacts on membrane lipid packing and especially on the cholesterol-sphingolipid-enriched raft domains with key roles in a number of signal transduction events [72,73]. Modification of membrane biophysical/biochemical properties by oxysterols thus most likely plays an important role in cytotoxicity elicited by these compounds (reviewed by Olkkonen and Hynynen, [74]). However, much of the cytotoxicity attributable to oxysterols is due to their ability to induce apoptosis, an aspect that has been extensively reviewed by Lordan *et al.* [11]. The two major apoptotic pathways are the mitochondrial (intrinsic) and the death receptor (extrinsic) pathways. Both pathways finish at the execution phase initiated by the activation of effector caspases, which in turn activate endonucleases degrading nuclear material and proteases that break down nuclear and cytoskeletal proteins. Caspase-3, -6, and-7 act as effector caspases at this stage, cleaving a variety of substrates such as poly(ADP-ribose) polymerase (PARP), resulting in the biochemical and morphological manifestations characterizing apoptotic cells [75]. Oxysterols can induce apoptosis by both pathways, and caspase-3, considered the most important effector caspase, is involved in apoptotic processes induced by a variety of oxysterols in different cell types (reviewed by Lordan *et al.*, [11]). However, the distinct oxysterols vary greatly in their ability to induce apoptosis, and also the pathways employed by distinct oxysterols are different. Therefore, there is most likely no universal mechanism responsible for oxysterol-induced apoptosis. When considering the cytotoxic activity of oxysterols, it is important to realize that most studies on the topic have been carried out by applying pure oxysterols on cultured cells. However, when oxysterols are administered as natural-like mixtures, as fatty acid esters, or as incorporated into lipoproteins, their cytotoxicity is strongly alleviated [76,77,78,79].

### 4.1. Death Receptor Pathway

Although there is only limited evidence to activation of the death receptor pathway by oxysterols, certain oxysterols are suggested to induce apoptosis via this route: Lee and Chau [80] demonstrated that 7β- and 25-OHC caused up-regulation of Fas and FasL (Fas ligand) and apoptosis of smooth muscle cells (SMCs) of vascular origin. This finding was supported by the study of Lordan *et al.* [81] showing that Fas inhibition reduced apoptosis of 7β-OHC-treated cells, and furthermore, that treatment with 7-KC predisposed human aortic SMC to Fas-mediated apoptotic cell death. 

Oxysterols have in certain cases connected also with tumor necrosis factor (TNF)-α induced apoptotic processes. Lee *et al.* [82] demonstrated that 7-KC treatment of SMCs resulted in TNF receptor-mediated cell death. It is to some extent unclear whether oxysterols in general have the capacity to induce TNF-α expression and secretion. There is evidence for such induction by 22-OHC and 7β-OHC in human monocytes and THP-1 macrophages [83,84]. However, there is also contradicting data from macrophages [85], and 7β-OHC or 7-KC induced apoptosis of human endothelial cells was not associated with enhanced TNF-α secretion [86].

### 4.2. Mitochondrial Pathway of Apoptosis

Alterations in mitochondrial transmembrane potential in response to various triggering factors result in the production of reactive oxygen species (ROS) or to mitochondrial membrane permeabilization. This permeabilization can be induced by the proapoptotic Bak/Bax proteins, the interaction of which with voltage-dependent anion channel/adenine nucleotide transporter results in the release of small molecules, such as cytochrome *c*, apoptosis-inducing factor (AIF), endonuclease G, and smac/DIABLO, activating both caspase-dependent and –independent cell death pathways [87]. Loss of mitochondrial membrane potential and release of cytochrome *c* upon oxysterol treatment has been documented in many studies (reviewed by Lordan *et al.*, [11]). It is important to note that different apoptotic pathways are induced dependent on the specific oxysterol(s) and the cell type employed; For instance, Ryan *et al.* [88] showed that in U937 cells, inhibition of cytochrome *c* release inhibited apoptosis induced by 7β-OHC but not by β-EPOX. Ghelli *et al.* [89] showed that in ECV304 cells treated with 7-KC no cytochrome *c* release was associated with the observed loss of cell viability. More mechanistic insight has been gained from recent studies: Kim and Lee [90] provided evidence that the tyrosine kinase inhibitor AG126 reduced 7-KC-induced cell death by suppressing mitochondrial permeability change, apparently via inhibition of ROS generation and GSH (glutathione) depletion. On the other hand, Gao *et al.* [91] demonstrated that apoptosis of J774 macrophage induced by the oxysterol cholesterol secoaldehyde (3β-hydroxy-5-oxo-5,6-secocholestan-6-al) is mediated via the mitochondrial pathway but without involvement of ROS. 

Initiation of mitochondrial apoptotic pathway may result from activation of the pro-apoptotic Bak/Bax proteins and concomitant inactivation of the anti-apoptotic Bcl-2 family proteins such as Bcl-2 and Bcl-xL [92]. While some studies have documented oxysterol impacts on the balance of cellular Bax vs. Bcl-2/Bcl-xL levels [93,94,95], no such effect was seen in 7β-OHC-treated Caco-2 cells [96], suggesting involvement of a Bax/Bcl-2 independent apoptotic process. Lordan *et al.* [97] presented evidence that increase of cytosolic Ca^2+^ concentration may be an early trigger of 7β-OHC-induced apoptosis in U937 cells—however, it did not seem to play a role in β-EPOX-induced apoptosis. These observations illustrate well the variability in the pathways of cell death induced by different oxysterols in different model systems employed. 

### 4.3. Protein Kinases Involved in Oxysterol-Induced Cell Death

A variety of protein kinases have been implicated in both the upstream induction phase of apoptosis and as targets of caspases during the actual execution of the apoptosis process, but also in other modes of cell death. The kinases implicated in apoptosis belong to mitogen activated protein kinase (MAPK), Akt/PKB, and the protein kinase C families (reviewed by Lordan *et al.*, [11]). Anticoli *et al.* [98] reported that 7-KC and 5,6-secosterol modulate differently the stress-activated MAPKs in liver cells: Pathologic concentrations of both oxysterols induced necrosis of the cells after 48 h treatment. 5,6-secosterol, but not 7-KC, induced cell senescence at high concentrations, but caused sustained ERK1/2 (extracellular signal activated kinases) activation and cellular proliferation at low concentrations. 7-KC was reported to induce apoptosis of PC12 neuroendocrine cells via ROS-mediated activation of NF-κB and Akt/PKB pathways [99]. Consistently, Liu *et al.* [100] found that lanthanum chloride suppresses cholestane-3β,5α,6β-triol induced apoptosis of ECV-304 cells via inhibition of ERK and NF-κB activation. Further, Laynes *et al.* [101] reported that inhibitors of p38 MAPK, ERK1/2 and JNK (Jun N-terminal kinase) suppressed the cytotoxicity of cholesterol secoaldehyde (ChSeco) in H9c2 cardiomyoblasts. ChSeco is known to induce apoptosis by both intrinsic and extrinsic pathways, and the above study suggested the involvement of ROS such as hydrogen peroxide in the ChSeco cytotoxicity.

## 5. Oxysterols in Inflammation and Immunity

### 5.1. Oxysterols as Immune Modulators

A number of reports have demonstrated the capacity of exogenously administered oxysterols, mainly 7-KC, 7β-OHC and 25-OHC, to enhance expression of inflammatory mediators, such as IL-8 and MCP-1, by macrophages [84,85,102] and other cell types not belonging to professional immune cells [86,103,104,105]. Importantly, endogenous 25-OHC has been shown to be secreted by dendritic cells and macrophages in response to Toll-like receptor activation, the cholesterol 25-hydroxylase (CH25H) gene expression being regulated by type I interferons via signaling through the interferon-α receptor and the JAK/STAT1 pathway [106,107]. The secreted 25-OHC was shown to suppress IgA class switching in B-cells, demonstrating a crucial function of endogenous 25-OHC in immunity control [106]. On the same theme, two recent studies identified the orphan G-protein coupled receptor EBI2 (Epstein-Barr virus induced gene 2) as a cell surface oxysterol receptor that directs B-cell migration [108,109]. 7α,25-dihydroxycholesterol generated from 25-OHC by oxysterol 7α-hydroxylase CYP7B1 was identified as the most potent ligand of this receptor. The data shows the potency of endogenous oxysterols as immune regulators, and brings up the idea that oxysterols or their synthetic analogues could offer therapeutic benefits either as adjuvants of immune modulators in the treatment of inflammation or autoimmune diseases. 

### 5.2. Inflammatory Functions and the LXRs

Increasing evidence demonstrates the ability of the LXRs, presumably as liganded by endogenous cellular oxysterols, to down-regulate inflammatory signaling (reviewed by Calkin and Tontonoz, [24]). Joseph *et al.* [23] first showed that LXR activation attenuated the expression of IL-6, iNOS (inducible nitric oxide synthase) and COX-2 (cycloxygenase 2) in macrophages stimulated with *E. coli* or lipopolysaccharide. Moreover, the LXR were shown to down-regulate the expression of matrix metalloproteinase 9 (MMP-9), a protease abundant in the macrophage-rich regions of atherosclerotic lesions and apparently plays a role in extracellular matrix degradation and plaque destabilization [23,110]. The anti-inflammatory effects of the LXRs have been attributed to their ability to dampen the activity of NF-κB, which controls expression of all of the genes mentioned above, via a mechanism designated transrepression [111]. More recent studies have demonstrated the cross-talk of LXRs with multiple Toll-like receptors (TLR): TLR3/4 stimulation was shown to suppress LXR signaling [112], and the LXRs were reported to dampen atherogenic signaling via the TLR2/TLR4/MyD88 route [113]. Moreover, activation of the macrophage LXRs by apoptotic cells was shown to enhance apoptotic cell clearance with simultaneous down-regulation of inflammatory signals [114]. Importantly, Bensinger *et al.* [115] also showed that LXR signaling couples the cellular sterol metabolism, via regulation of ABCG1 (cholesterol transporter) expression, with T-cell proliferation in acquired immune response. These investigations have revealed that the LXRs control of number of immune and inflammatory pathways that play important roles in the development of atherosclerosis, and underscore that potential value of the LXRs as future therapeutic targets.

## 6. Niemann-Pick C1 Protein and StarD5 Bind Both Cholesterol and 25-OHC

Niemann-Pick C (NPC) disease is characterized by accumulation of free cholesterol and sphingolipids within late endocytic compartments of cells [116]. The disease is caused by mutations in either of two proteins designated NPC1 or NPC2. NPC1, mutations of which account for a majority of the disease, is a multi-spanning membrane protein localized in late endosomes, and has a consensus cholesterol binding motif consisting of five trans-membrane helices. The mechanism by which NPC1 facilitates egress of lipids from late endocytic compartments is poorly understood, but it seems to receive cholesterol from the NPC2 protein localizing in endosome lumen [117] and was recently suggested to donate it to the OSBP homologue ORP5 (see section 7; [118]). Interestingly, NPC1 was found to bind not only cholesterol but also 25-OHC, the binding of which was efficiently competed by 24(S)-OHC and 27-OHC [119]. The authors found that cholesterol and 25-OHC bind not to the trans-membrane helix motif but to a luminal loop of NPC1 [120]. However, in further experiments it seemed that the oxysterol binding may not be involved in the classical function of NPC1 in mediating cholesterol transport. 

StarD5, a cytoplasmic protein belonging to the family of proteins carrying a steroidogenic acute regulatory protein (StAR)-related lipid transfer (START) domain [121], was shown to bind both cholesterol and 25-OHC [122]. Although the physiologic function of StarD5 is poorly understood, it was shown to enhance steroidogenesis in an *in vitro* assay, evidencing for its ability to transfer cholesterol [123]. As for NPC1, the functional role of 25-OHC binding by StarD5 remains unclear.

## 7. The Cytoplasmic Oxysterol-Binding Proteins—Functions in Lipid Metabolism and Sterol-Dependent Signaling

Families of proteins with sequence homology to the cytoplasmic oxysterol-binding protein, OSBP, are present throughout the eukaryotic kingdom. In mammals the family consists of 12 members. These proteins, designated OSBP-related (ORP) or OSBP-like (OSBPL) proteins, have been implicated in a variety of functions involving cellular lipid metabolism, vesicle transport, and cell signaling (reviewed by Vihervaara *et al.*, [124]). However, the mechanisms of their action have remained poorly understood. Furthermore, many family members have, in addition to oxysterols, been found to bind cholesterol, which is far more abundant in cells. Hence, the identity of the true physiologic ligands of the ORP proteins is not fully solved. In the following we summarize the most important findings on ORP function.

### 7.1. OSBP Regulates Cellular Lipid Homeostasis

OSBP, the founder member of the ORP family, acts as a sterol sensor involved in the regulation of sphingomyelin synthesis via control of the localization and activity of PtdIns-4-kinase IIα and ceramide transporter, CERT, responsible for non-vesicular transport of ceramides from the endoplasmic reticulum (ER) to Golgi where sphingomyelin synthase is located [125,126]. Adenoviral overexpression of rabbit OSBP in mouse liver was shown to result in an increase of plasma very-low-density lipoprotein (VLDL) and liver tissue triglycerides (TG) [127]. Analysis of the underlying mechanism revealed up-regulation of *SREBP-1c* expression and increase of the active nuclear form of this lipogenic transcription factor in the liver of mice injected OSBP-expressing adenovirus. Silencing of OSBP by RNA interference in cultured hepatocytes attenuated the insulin induction of *SREBP-1c* and fatty acid synthetase (*FAS*), as well as TG synthesis. Furthermore, OSBP overexpression was shown to inhibit phosphorylation of the ERKs. In the light of the finding that changes in ERK activity impact the stability of nuclear SREBP-1c [128], this provides one tentative mechanistic explanation to the OSBP overexpression phenotype: Its impact of ERK could modify the stability of nuclear SREBP-1c and thereby hepatic lipogenesis and VLDL secretion. The findings demonstrate a role of OSBP as a sterol-dependent regulator sphingomyelin synthesis and hepatic TG metabolism, as well as a putative function in insulin-induced signaling cascades.

Bowden and Ridgway [129] reported that silencing of OSBP by RNA interference resulted in increased cellular amount and cholesterol efflux activity of ABCA1, in the absence of effects on the *ABCA1* mRNA level or LXR activity. OSBP knock-down was shown to increase the half-life of the ABCA1 protein, the effect being dependent on an intact OSBP sterol-binding domain. Thus, it seems that OSBP opposes the activity of LXR by destabilizing ABCA1.

### 7.2. Roles for OSBP in ERK and JAK-2/STAT3 Signaling, Amyloid Precursor Protein Processing, and Hepatitis C Virus Assembly

R. Anderson’s group (Wang *et al.* [130,131]) identified OSBP as a sterol-sensing scaffolding factor that regulates the dephosphorylation and hence the activity of the ERK, important components of the mitogen activated protein kinase (MAPK) signaling pathways. Romeo and Kazlauskas [132] found that up-regulation of profilin-1, an actin-binding protein implicated in endothelial dysfunction and atherosclerosis, by 7-KC is mediated by OSBP. The signaling route involves interaction of the OSBP-7-KC complex with the tyrosine kinase JAK-2, which phosphorylates Tyr394 on OSBP. This apparently leads to the activation of STAT3, which induces profilin. An important implication of these findings is that also other members of the ORP family could act as lipid sensors with scaffolding functions in cell signaling. Consistent with this idea, Lessman *et al.* [133] demonstrated that ORP9 contains a phosphoinositide-dependent kinase-2 (PDK-2) phosphorylation site, the phosphorylation of which is dependent on PKC-β or mTOR. ORP9 was shown to interact with these kinases to negatively regulate phosphorylation of the PKD-2 site in Akt/protein kinase B, a major controller of cell survival, cell cycle progression, and glucose metabolism. 

Zerbinatti *et al.* [134] demonstrated that OSBP overexpression in a neuroglioma cell line and in HEK293 cells down-regulated the processing of amyloid precursor protein (APP) to β-amyloid (Aβ); OSBP silencing had the opposite effect. OSBP overexpression resulted in the sequestration of APP-Notch2 heterodimers in the Golgi complex, an effect reversed by addition of the OSBP high-affinity ligand 25-OHC. This is consistent with the established findings that the distribution of OSBP itself between cytosol/ER and Golgi membranes is regulated by the cellular sterol status [135,136], and suggests that OSBP modulates the intracellular trafficking of APP. OSBP could thus play a pivotal role in controlling APP metabolism in a sterol-dependent fashion. 

Localization of OSBP in the Golgi complex is negatively regulated by protein kinase D (PKD)-mediated phosphorylation [137]. OSBP was shown to interact in the Golgi complex with the Hepatitis C virus (HCV) non-structural protein NS5A, and OSBP silencing inhibited secretion of HCV particles by infected cells [138]. Moreover, HCV release was shown to be suppressed by PKD, an effect mediated by phosphorylation of OSBP and its functional partner, CERT [139]. A further implication of OSBP-mediated lipid regulation of microbial infection is provided by the study of Auweter *et al.* [140] suggesting that OSBP enhances *Salmonella* replication. As a conclusion, there is increasing evidence for pleiotropic functions of OSBP as a modulator of signaling and intracellular transport events. These properties make OSBP a target that is highjacked by micro-organisms to facilitate their intracellular replication and progeny release. 

### 7.3. ORP8 and the Transcriptional Control of Lipid Metabolism

Related with the above report on OSBP, Yan *et al.* [141] demonstrated that silencing of ORP8 expression in THP-1 macrophages induces the transcription of *ABCA1* and, consequently, cholesterol efflux to apolipoprotein A-I. This effect was reproduced using a luciferase reporter assay, in which ORP8 silencing synergized with a synthetic LXR agonist and was significantly suppressed when a mutant *ABCA1* promoter devoid of a functional LXR response (DR4) element was used, providing the first solid piece of evidence for a functional interplay between the LXR and the ORPs. Importantly, ORP8 was found to be abundant in the macrophages of human coronary artery lesions, bringing up the possibility that ORP8 may play a role, possibly an adverse one, in the development of atherosclerotic lesions. Our recent work employing mouse macrophage models indicates that the modulation of ABCA1 expression upon ORP8 silencing or knock-out is weak and is most likely mediated by an indirect mechanism (unpublished observations).

We recently found that ORP8 overexpression in mouse liver results in a reduction of plasma and liver tissue lipid levels, associated with down-regulation of the active, nuclear forms of SREBP- 1 and -2 [142]. Moreover, ORP8 was found to physically interact with the nuclear pore complex component NUP62, and a normal level of NUP62 in HuH7 hepatoma cells was found to be necessary for the ORP8-mediated reduction of nuclear SREBPs. These findings indicated that ORP8 has the capacity to modulate SREBP-dependent transcription, and it may affect the activity of several transcription factors systems, possibly via an indirect mechanism involving the nuclear pore complex and transport of transcription factors and other nuclear components in and out of the nucleus.

### 7.4. ORP1L Regulates Late Endosome Motility and Macrophage Lipid Metabolism

Johansson *et al.* [143] employed ORP1L knock-down to show that the protein is required for the clustering of late endocytic compartments in the pericentriolar region. The protein was shown to form a complex with the late endosomal GTPase Rab7 and its second effector protein RILP. The tripartite complex apparently recruits dynein/dynactin motor to late endosome (LE) membranes to drive minus-end directed motility of the compartments. In a recent study we demonstrated how ligand interactions regulate ORP1L function [144]. By deleting four amino acids from the lid of ORP1L ORD we created a mutant deficient in binding oxysterols (ORP1L Δ560−563). This mutation did not result in translocation of the protein from the LE, but the distribution of LE changed dramatically. Whereas the wild-type (WT) protein induced LE clustering, the LE decorated by mutant ORP1L displayed a scattered phenotype. Similar LE scattering was recently achieved by cellular cholesterol depletion, and ORP1L was shown to be key mediator of the effect [145]. A double mutant deficient in sterol binding and containing a disrupted ER-targeting two phenylalanines in an acidic tract (FFAT) motif (ORP1L Δ560−563 mFFAT) induced LE clustering, demonstrating that destruction of the ER interaction of ORP1L reversed the scattered LE phenotype [144]. A model arising from these experiments and those of Rocha *et al.* [145] is that non-sterol bound ORP1L bridges between Rab7 on LE and VAPs in the ER, thus forming a membrane contact site (see Levine and Loewen, [146]), which is likely to restrict LE motility; Silencing of ORP1L increased the motility of LE and late endosomal tracks had a more peripheral distribution, probably due to impairment of dynein/dynactin recruitment, or to an altered balance between the minus- and plus-end directed motor complexes 

Yan *et al.* [147] showed that transgenic macrophages overexpressing ORP1L increased the size of atherosclerotic lesions in LDL receptor deficient mice. The transgenic macrophages displayed a defect in cholesterol efflux to spherical high-density lipoproteins (HDL) and reduced expression of ABCG1 and apolipoprotein E, as well as increased expression of phospholipid transfer protein (PLTP). All these genes are subject to transcriptional regulation by the LXRs. Furthermore, ORP1L overexpression in cultured macrophages was shown to attenuate the response of the *ABCG1* mRNA to the LXR agonist 22(R)OHC, which is also a ligand of ORP1L. The work of Vihervaara *et al.* [144] showed that ORP1L silencing in mouse Raw264.7 macrophage inhibited the efflux of endocytosed lipoprotein cholesterol to apolipoprotein A-I, thus presumably affecting ABCA1-dependent cholesterol transport. One interpretation of these results is that ORP1L could modulate the LXR ligand interactions, thereby affecting the expression of LXR target genes and the development of atherosclerosis. However, it is also possible that other, more indirect mechanisms account for the observed phenotypic effect. 

### 7.5. Sterol Transporter Function of ORPs

Evidence for ORP function in actual sterol transport was reported by the groups of W. Prinz and A. Menon: Raychaudhuri *et al.* showed that the *Saccharomyces cerevisiae* ORP Osh4p is able to extract sterols from liposomes and to deliver them to acceptor membranes. Cells deficient of all seven Osh proteins displayed a marked, 80% reduction in the transfer of cholesterol or ergosterol from plasma membrane (PM) to ER [148]. Involvement of Osh proteins was also suggested in transfer of newly synthesized ergosterol from ER to PM; depletion of all 7 Osh proteins reduced the rate of this transport 5-fold [149]. However, the group of A. Menon recently reached the opposite conclusion: They provided evidence that the role of Osh proteins in ER to PM ergosterol transport is minor, and that Osh protein defect rather distorts the sterol organization in the plasma membrane [150]. 

Of the mammalians ORPs, OSBP, ORP9L, and the ORP5 ORD have been shown to transfer cholesterol between membranes *in vitro* [118,151]. Silencing of ORP5, which is anchored to ER membranes, was shown to cause cholesterol accumulation to the limiting membrane of late endosomes. The protein was demonstrated to associate with NPC1, consistent with a model in which ORP5 accepts cholesterol from NPC1 and routes it to the ER [118]. Jansen *et al.* [152] recently provided evidence that mammalian ORPs have the capacity to stimulate cholesterol transfer in live cells. They used an assay to monitor the transport of a Bodipy-labeled cholesterol derivative from the PM to lipid droplets (LD), and studied the contribution of ORP overexpression to the process. In this study, out of all human ORPs, overexpression of two ORPs of the short subtype, ORP1S and ORP2, gave the largest increase in sterol transport, and silencing both of them simultaneously decreased the transport rate [152]. The effect of the ORPs was further localized to the PM-ER transport step, whereas the rapid ER-LD sterol transport was unaffected by ORP manipulation. In addition, a functional sterol-binding pocket was shown to be necessary for the observed transport enhancement in the case of ORP2. The finding that the PM to ER sterol transfer was most affected by the short ORPs, ORP1S and ORP2, could reflect the ability of these proteins, devoid of PH domains, to associate and dissociate from membranes more rapidly than the long ORPs, perhaps allowing more efficient shuttling between donor and acceptor membranes. This study supports a role for ORPs in sterol transfer but like the studies on yeast Osh proteins, does not provide conclusive evidence for a sterol carrier function. One cannot exclude the possibility that the role of ORP sterol binding might be regulatory, and the observed impacts on sterol trafficking indirect, for instance via the control of membrane contact site formation or the function of other proteins at such sites (reviewed in Levine and Loewen, [146]). Alternatively, modulation of membrane lipid composition by ORPs could affect the ability of membranes to accommodate sterols and thereby alter sterol fluxes, as suggested by Georgiev *et al.* [150]. 

### 7.6. ORP3 Regulates Cell Adhesion

ORP3 and ORP7 were found to interact physically with R-Ras, a small GTPase that regulates cell adhesion and migration [153], implying a role of these ORPs in R-Ras signaling. Lehto *et al.* [154] reported that ORP3 controls cell adhesion and spreading, organization of the actin cytoskeleton, β1-integrin activity and macrophage phagocytic function, cellular processes also subject to regulation by R-Ras. Furthermore, ORP3 phosphorylation was suggested to be regulated by outside-in signals mediated by integrins and cadherins. ORP3 is expressed abundantly in leukocytes and in several epithelia, and its abnormally high expression is detected in certain forms of leukemia and solid tumors, suggesting that the protein may modify cell signaling and adhesion properties in a manner that facilitates malignant growth.

### 7.7. S. cerevisiae Osh4p Modulates the Golgi Phosphatidylinositol-4-Phosphate Pool and Secretory Vesicle Transport

Deletion of the *S. cerevisiae* ORP *OSH4/KES1* leads to by-pass of the temperature-sensitivity of mutants in *SEC14*, a gene encoding a phosphatidylinositol transfer protein (PITP; Sec14p) essential for secretory vesicle biogenesis [155,156]. Osh4p thus acts as a negative regulator of Golgi secretory function, but the underlying mechanism has remained poorly understood. The group of C. McMaster showed that Osh4p reduces both the cellular content of phosphatidylinositol-4 phosphate (PI4P) and its availability for recognition by other proteins, which include components required for transport vesicle formation [157]. Interestingly, Alfaro *et al.* [158] recently found a direct role for Osh4p in exocytic vesicle transport: They showed that Osh4p associated with exocytic vesicles that move from the mother cell into the bud, where Osh4p facilitated vesicle docking by the exocyst tethering complex at sites of polarized growth on the plasma membrane. Osh4p formed complexes with the small GTPases Cdc42p, Rho1p and Sec4p, and the exocyst complex subunit Sec6p, which was also required for Osh4p association with vesicles. Although Osh4p directly affected polarized exocytosis the relationship of this function with that in sterol trafficking remained unclear. Importantly, de Saint-Jean *et al.* [159] recently made the ground-breaking finding that Osh4p, in addition to sterols, binds also PI4P employing the same binding pocket. It is able to exchange bound sterol for PI4P and transport the two lipids between membranes along opposite routes. The results suggest a model in which Osh4p transports sterol from the ER to late compartments and, in turn, PI4P backward from trans-Golgi and plasma membrane to the ER. This transport cycle would create a sterol gradient, promoting sterol enrichment in membranes of the late secretory pathway, and play a role in controlling the concentration and distribution of PI4P, with an essential function in secretory vesicle transport.

## 8. Future Perspectives

**Figure 2:**
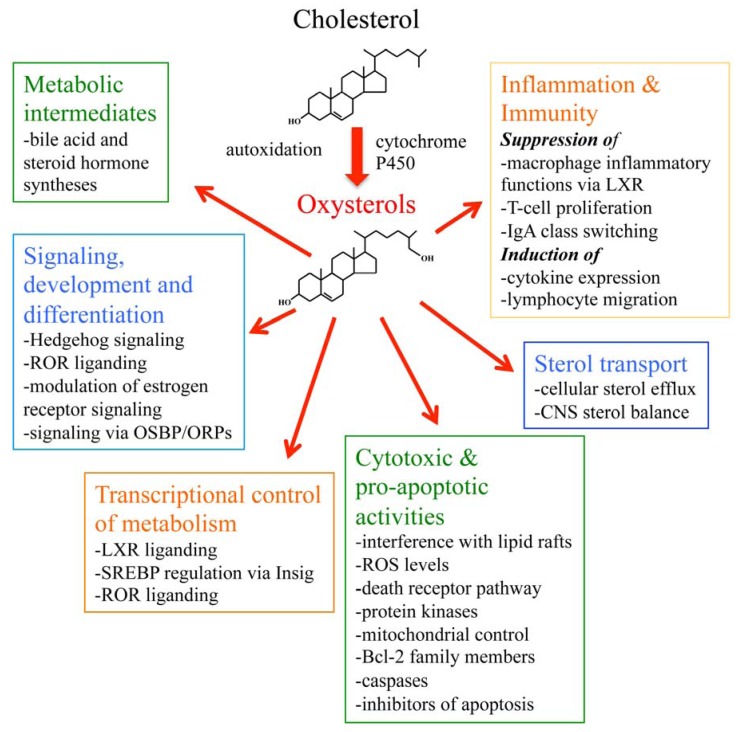
A schematic presentation summarizing the major functions of oxysterols. ROR, retinoic acid receptor-related orphan receptor; OSBP, oxysterol-binding protein; ORP, OSBP-related protein; LXR, liver X receptor; SREBP, sterol regulatory element binding protein; Insig, insulin-induced gene; ROS, reactive oxygen species; Bcl-2, B-cell lymphoma 2; IgA, immunoglobulin A; CNS, central nervous system.

Oxysterols were previously considered to represent harmful substances that accumulate in pathophysiologic states. However, work carried out during the past decade has revealed their physiologic functions as signaling molecules that maintain cellular and body lipid homeostasis and determine cell fate. The discovery of oxysterol interactions with new receptors involved in lipid metabolism, such as the LXRs, Insigs, RORs, the estrogen receptors, NPC1, StarD5 and the OSBP-related proteins, paves the way for future work to elucidate in detail the mechanisms of oxysterol action in lipid metabolism and in diseases such as atherosclerosis and Alzheimer’s disease. Discovery of oxysterols as regulators of Hedgehog signaling uncovered their role in embryonic development, and the recent finding on oxysterol liganding of EBI2 pinpoints a novel effector route for regulation of immunity. These observations have shed light on the diversity of oxysterol activities and opened new perspectives in oxysterol research: With developing oxysterol analytical methodologies and advanced approaches of *in vivo* genetic manipulation of various organisms and cells in culture, we can expect a plethora of novel connections of oxysterols and their cellular receptors regimes such as differentiation, development, and immunity. The currently known major functions of oxysterols are summarized in Figure 2. The major functions of cellular oxysterol receptors are summarized in Table 2.

**Table 2:** Cellular receptors for oxysterols.

Receptor (or rec. family)	Function	References
LXRα, LXRβ	Transcriptional regulation of cholesterol adsorption and cellular efflux, cholesterol and bile acid synthesis, neutral lipid secretion into bile, inflammation and immune response	[22*,^24^*]
OSBP/ORPs	Regulation of lipid homeostasis, vesicle transport and cell signaling	[124*]
Insig	Regulation of SREBP maturation; cholesterol and fatty acid biosynthesis and LDL receptor expression	[36,37]
StarD5	Cellular cholesterol metabolism and transport; up-regulated upon ER stress	[121*,^122^]
NPC1	Egress of endocytosed cholesterol out of late endocytic compartments	[119,120]
RORα, RORγ	Transcriptional regulation of genes involved in development. metabolism, and immunity	[52,53]
EBI2/GPR183	Control of B-cell migration	[108,109]
Smoothened	Hedgehog signaling	[63]

* The indicated references are review articles included to avoid excessive listing of literature
